# The Effects of Time Window-Averaged Mobility on Effective Reproduction Number of COVID-19 Viral Variants in Urban Cities

**DOI:** 10.1007/s11524-022-00697-5

**Published:** 2022-11-29

**Authors:** Sachiko Kodera, Keigo Hikita, Essam A. Rashed, Akimasa Hirata

**Affiliations:** 1 Department of Electrical and Mechanical Engineering, Nagoya Institute of Technology, Nagoya, 466-8555 Japan; 2 Graduate School of Information Science, University of Hyogo, Kobe, 650-0047 Japan; 3 Center of Biomedical Physics and Information Technology, Nagoya Institute of Technology, Nagoya, 466-8555 Japan

**Keywords:** COVID-19, Effective reproduction number, Mobility, Transmission model

## Abstract

During epidemics, the estimation of the effective reproduction number (ERN) associated with infectious disease is a challenging topic for policy development and medical resource management. The emergence of new viral variants is common in widespread pandemics including the severe acute respiratory syndrome coronavirus 2 (SARS-CoV-2). A simple approach is required toward an appropriate and timely policy decision for understanding the potential ERN of new variants is required for policy revision. We investigated time-averaged mobility at transit stations as a surrogate to correlate with the ERN using the data from three urban prefectures in Japan. The optimal time windows, i.e., latency and duration, for the mobility to relate with the ERN were investigated. The optimal latency and duration were 5–6 and 8 days, respectively (the Spearman’s *ρ* was 0.109–0.512 in Tokyo, 0.365–0.607 in Osaka, and 0.317–0.631 in Aichi). The same linear correlation was confirmed in Singapore and London. The mobility-adjusted ERN of the Alpha variant was 15–30%, which was 20–40% higher than the original Wuhan strain in Osaka, Aichi, and London. Similarly, the mobility-adjusted ERN of the Delta variant was 20%–40% higher than that of the Wuhan strain in Osaka and Aichi. The proposed metric would be useful for the proper evaluation of the infectivity of different SARS-CoV-2 variants in terms of ERN as well as the design of the forecasting system.

**Supplementary Information:**

The online version contains supplementary material available at 10.1007/s11524-022-00697-5.

## Introduction

After the coronavirus disease-2019 (COVID-19) outbreak, the everyday routine has been dramatically influenced. In the early period of the COVID-19 pandemic, a shortage of medical resources was reported, including its allocation, although some policies, such as city lockdown, have been conducted [1]. After the discovery and administration of vaccinations, the number of new daily positive cases (DPC) has notably decreased [2]. The first country to reach a high vaccination rate was Israel, which peaked in mid-January 2021. Conversely, its DPC later increased after July and then approach another peak in early September [3]. This is likely attributable to the widespread of the new Delta variant (severe acute respiratory syndrome coronavirus 2 (SARS-CoV-2) B.1.617 lineage) whose infectivity would be higher than those of the original Wuhan strain [4].

With the appearance of new viral variants, their infectivity was one of the concerns in the estimation of medical resources allocation to control potential widespread morbidity. However, its straightforward comparison is difficult since different contributing factors exist, including policy [5, 6], human behavior [7], and environmental conditions [8, 9]. A potential useful approach is to acquire the knowledge and experience from other countries toward an appropriate and timely policy decision.

In the epidemic theory, the basic reproduction number is used to measure the transmission potential of a disease [10]. This is an appropriate index when discussing the viral infectivity, because it represents the average number of secondary infections produced by a typical case of an infection in a population under the assumption where everyone is susceptible. However, in the real world, the whole population would not be totally susceptible to an infection after the waves of COVID-19. In addition, prevention measures were taken in most countries. Thus, not all contacts would result in infection. In this situation, the effective reproduction number (ERN) would be more realistic index, which is defined as the expected number of secondary cases arising from a single primary case. Several studies investigated ERN for different factors [11–13], but their definitions are not always identical [14, 15]. Estimating the new DPC or ERN is not a straightforward task due to the incubation time of the infection, in addition to the latency between the actual infection incident time and reporting of infection in healthcare facilities. One of the reasons for this difficulty is attributable to public mobility [15].

Many models were developed for forecasting the future of COVID-19 have been provided [16–19]. In [19], the proportion of people that have seen media broadcast about COVID-19 was considered as the index of social distancing. Our previous study revealed that DPC for two weeks in the future can be estimated using a machine learning (one of the categories of artificial intelligence) approach with an accuracy of 81.6% [20]. The input data include the mobility change at different urban locations (retail and recreation, grocery and pharmacy, parks, transit stations, etc.), weather data, and labels retributed to other associated factors. The dominant influencing factor of the new DPC was the mobility at the transit stations [20]. The same tendency was observed in other countries. The growth rate of COVID-19 has a positive correlation with the mobility during the period of the DPC upsurge (increase cases in COVID-19 wave) in China [21]. The prediction based on the mobility was proposed [22]. To address the impact of urban mobility on COVID-19 propagation, the traffic systems have been developed [23].

A major characteristic of machine learning architecture is the well-known black-box feature, which is more explainable in terms of nonlinear regression with a relatively large number of parameters compared to logistic regression. However, one drawback of the machine learning approach is that the mechanism cannot be explained straightforwardly for easier implementation and data process tracking. Thus, a straightforward interpretation should be further explored following the above findings (see “Data” section).

Considering the incubation time of the epidemic, the latency effect cannot be ignored. The single factor, the mobility at the transit stations, explains the DPC (accuracy is more than 80%) in six urban prefectures [20], thus the role of this factor should be analytically assessed considering the latency and interval. Comprehensively, no earlier study discussed the importance of mobility considering the latency and time window size effects.

The present study aimed to explore a simple approach to surrogate ERN values from public mobility and discuss the difference of ERN for different viral variants (Wuhan strain, Alpha, and Delta variants). One open question is the extent of differences observed between different COVID-19 variants for given public mobility, which is rather essential in public awareness than ERN estimation. The major novelty of this study is the contribution of time window and latency (averaging interval) of mobility in the ERN (see “Effective Reproduction Number” section).

## Materials and Methods

### Data

The three prefectures chosen in this study include Tokyo, Osaka, and Aichi, whose populations are ranked first, third, and fourth in Japan, respectively. The second-largest population is Kanagawa prefecture, but it is adjacent to Tokyo, thus it is not considered in this study. The following external cities were selected to confirm the tendency in Japan: London and Singapore, which have almost the same volume of the total population as the three prefectures and the primary public transportation characterized at train stations.

Data collection started from February 15, 2020, to November 31, 2021. Concerning the situation in Japan, one of the features is the initially mild spread of COVID-19, thus vaccination was retarded by a few months compared to that in European and North American countries. Thus, the effects of vaccination were marginal except for those of healthcare professionals until June 2021, in which the Alpha variant (SARS-CoV-2 B.1.1.7 lineage) was dominant [24]. Therefore, we decided to use the acquired data from different regions in Japan to demonstrate the validity of the proposed metric. Public movements were estimated from Google mobility reports (https://www.google.com/covid19/mobility/) that represented global data records from February 15, 2020. Google mobility represents the percentage of change from baseline at spots defined as retail and recreation, grocery and pharmacy, parks, transit stations, workplaces, and residential. Baseline is defined as the median values for five weeks from January 3 to February 6, 2020.

The numbers of confirmed COVID-19 DPC were obtained from online open data sources provided by the Japanese Ministry of Health, Labor, and Welfare (https://www.mhlw.go.jp/stf/covid-19/kokunainohasseijoukyou.html). The number of days from sample correction to healthcare facility reporting is usually 0.5–2 days in Japan [25]. The DPC in Singapore was obtained from an online open data source at Our World in Data (https://ourworldindata.org/). The DPC in London was obtained from an online open data source at the GOV.UK (https://coronavirus.data.gov.uk/).

**Fig. 1:**
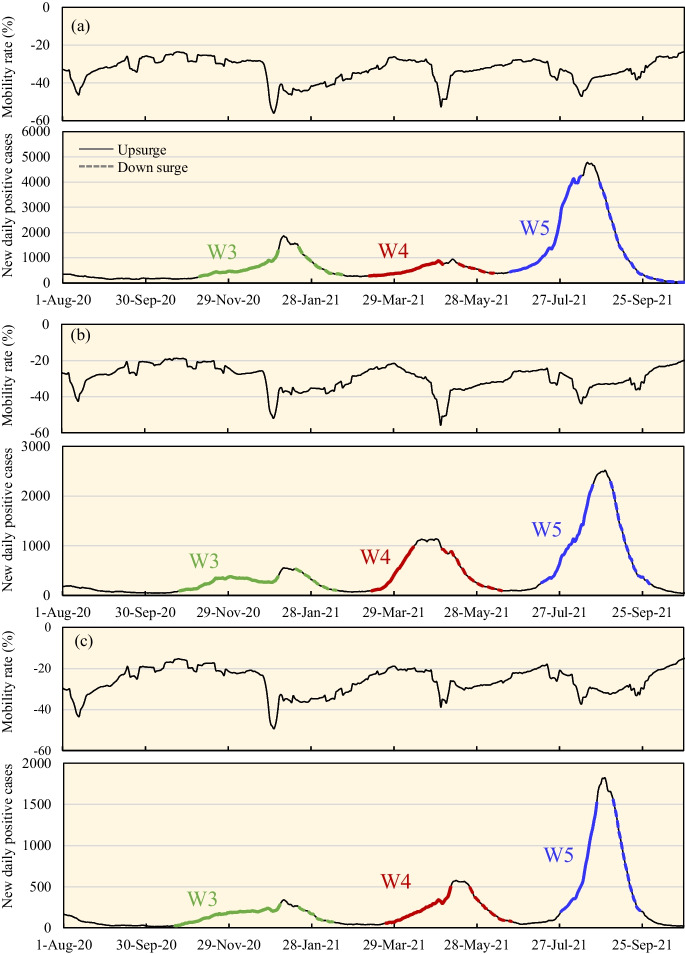
**a**–**c** Mobility change (%) at transit stations (upper) and daily confirmed new positive cases (lower) in **a** Tokyo, **b** Osaka, and **c** Aichi prefectures. The colored lines show the spread duration of the third, fourth, and fifth pandemic waves. All lines represent 7-day averages

Figure 1 summarizes the new DPC and mobility at the transit stations for three prefectures in Japan. Japan had five pandemic waves from February 2020 to October 2021. The stages of the spread were determined from 10 to 90% of the peak values in each wave, the same as that in our previous study [26]. The exact definition for the period of the third (W3), fourth (W4), and fifth (W5) waves in the three prefectures is defined as listed in Supplementary Materials. For each wave, the Wuhan strain, Alpha, and Delta variants were respectively dominant [27].

### Effective Reproduction Number

The ERN (*R*_*t*_) was computed using the following equation [28]:

1 $$R_{t} = \left( {{{\sum\limits_{i = 1}^{s} {DPC_{t - i} } } \mathord{\left/ {\vphantom {{\sum\limits_{i = 1}^{s} {DPC_{t - i} } } {\sum\limits_{i = s + 1}^{2s} {DPC_{t - i} } }}} \right. \kern-\nulldelimiterspace} {\sum\limits_{i = s + 1}^{2s} {DPC_{t - i} } }}} \right)^{{{\mu \mathord{\left/ {\vphantom {\mu s}} \right. \kern-\nulldelimiterspace} s}}} ,$$

where *s* = 7 is the number of days for a specific period and *μ* = 5 (days) is the mean latency after the infection. In the following discussion, we use mobility-adjusted ERN as one of the metrics. This is used for the comparison of ERN compensating for the effect of mobility, enabling a proper comparison of viral infectivity.

### Averaging Time Windows of Mobility

In this study, for simplicity, the mobility at the transit stations was considered as a surrogate to represent COVID-19 transmission to avoid nonlinear regression computations. This is based on our previous study results that revealed the mobility at the transit stations (Google mobility) as the most important factor characterizing the new DPC [29]. When using machine learning, the accuracy of the 2-week new DPC forecasting is > 82.6%, whereas the remaining factors included the weather and condition of state-of-emergency [20]. Thus, a time window averaging of the mobility was investigated, which is approximately characterized by the incubation time and latency from sample collection to reporting in healthcare facilities to relate with the ERN [20]. The mobility at transit stations was averaged over time windows (days) considering the latency (days) (e.g., setting the duration to 6 days and latency to 4 days means averaging the mobility of 4–9 days before the relevant date).

The correlation between the ERN and public mobility with latency was analyzed using the Pearson and Spearman rank correlation. The JMP software package (SAS Institute, Cary, NC, USA) was used for statistical analysis. A *p*-value of < 0.05 was considered statistically significant to specify the dominant factors that influence the rates.

## Results

**Fig. 2:**
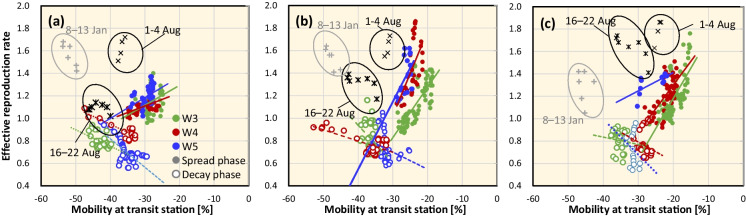
Correlation between the mobility at the transit station and the effective reproduction rate in **a** Tokyo, **b** Osaka, and **c** Aichi. Mobility was averaged over 8 days with a latency of 6 days. Green, red, and blue correspond to the third, fourth, and fifth waves shown in Fig. 1, respectively

Table 1 shows the relationship between ERN and averaged mobility at transit stations over different time windows with different latency setups. The ERN during long vacations, such as the New Year’s holiday season, summer holidays, consecutive holidays, etc., were excluded because of the tendency of different corresponding mobility than those of weekdays (Fig. 2). As shown in Table 2, the optimal duration and latency were different for different pandemic waves. A weaker correlation was observed in Tokyo (*R*^2^ was 0.109, 0.512, and 0.235 for W3, W4, and W5, respectively) than those in Osaka (*R*^2^ was 0.607, 0.603, and 0.365 for W3, W4, and W5, respectively) and Aichi (0.524, 0.317, and 0.631 for W3, W4, and W5, respectively). The coefficient of correlation was similar and statistically significant in 6–8 days for the duration and 6–7 days for the latency. These values were chosen as 8 and 6 days, respectively, so that the averaged *R*^2^ becomes the maximum for the three waves in the three prefectures.

**Table 1:** Coefficient of determination for the correlation between the effective reproduction numbers and the average mobility at transit stations over different durations and latencies in (a) Tokyo, (b) Osaka, and (c) Aichi

Time windows (days)	Latency (days)	W3			W4			W5		
		*R*^2^	*p*-value		*R*^2^	*p*-value		*R*^2^	*p*-value	
(a)										
6	4	0.098	0.017	(*)	0.261	< 0.0001	(***)	0.005	0.666	(-)
	5	0.109	0.011	(*)	0.307	< 0.0001	(***)	0.059	0.121	(-)
	6	0.079	0.032	(*)	0.316	< 0.0001	(***)	0.119	0.025	(*)
	7	0.035	0.158	(-)	0.333	< 0.0001	(***)	0.160	0.009	(**)
	8	0.006	0.552	(-)	0.369	< 0.0001	(***)	0.208	0.002	(**)
7	4	0.087	0.025	(*)	0.286	< 0.0001	(***)	0.014	0.459	(-)
	5	0.106	0.013	(*)	0.334	< 0.0001	(***)	0.076	0.078	(-)
	6	0.076	0.036	(*)	0.343	< 0.0001	(***)	0.152	0.011	(*)
	7	0.040	0.135	(-)	0.370	< 0.0001	(***)	0.190	0.004	(**)
	8	0.010	0.145	(-)	0.420	< 0.0001	(***)	0.235	0.001	(**)
8	4	0.085	0.026	(*)	0.316	< 0.0001	(***)	0.024	0.325	(-)
	5	0.102	0.015	(*)	0.358	< 0.0001	(***)	0.103	0.039	(*)
	6	0.077	0.035	(*)	0.377	< 0.0001	(***)	0.177	0.006	(**)
	7	0.043	0.120	(-)	0.416	< 0.0001	(***)	0.213	0.002	(**)
	8	0.021	0.280	(-)	0.467	< 0.0001	(***)	0.204	0.003	(**)
9	4	0.084	0.028	(*)	0.341	< 0.0001	(***)	0.042	0.194	(-)
	5	0.104	0.014	(*)	0.387	< 0.0001	(***)	0.125	0.022	(*)
	6	0.079	0.032	(*)	0.418	< 0.0001	(***)	0.199	0.003	(**)
	7	0.056	0.073	(-)	0.458	< 0.0001	(***)	0.192	0.004	(**)
	8	0.035	0.162	(-)	0.512	< 0.0001	(***)	0.157	0.009	(**)
(b)										
6	4	0.518	< 0.0001	(***)	0.523	< 0.0001	(***)	0.344	0.007	(**)
	5	0.587	< 0.0001	(***)	0.446	< 0.0001	(***)	0.298	0.013	(*)
	6	0.584	< 0.0001	(***)	0.357	0.0004	(***)	0.312	0.011	(*)
	7	0.517	< 0.0001	(***)	0.276	0.002	(**)	0.232	0.032	(*)
	8	0.438	< 0.0001	(***)	0.227	0.007	(**)	0.110	0.154	(-)
7	4	0.551	< 0.0001	(***)	0.603	0.000	(***)	0.365	0.005	(**)
	5	0.607	< 0.0001	(***)	0.478	< 0.0001	(***)	0.346	0.006	(**)
	6	0.596	< 0.0001	(***)	0.379	0.0002	(***)	0.292	0.014	(*)
	7	0.533	< 0.0001	(***)	0.316	0.001	(**)	0.243	0.027	(*)
	8	0.463	< 0.0001	(***)	0.291	0.002	(**)	0.200	0.048	(*)
8	4	0.566	< 0.0001	(***)	0.510	< 0.0001	(***)	0.362	0.005	(**)
	5	0.605	< 0.0001	(***)	0.415	< 0.0001	(***)	0.265	0.020	(*)
	6	0.587	< 0.0001	(***)	0.353	0.0004	(***)	0.216	0.039	(*)
	7	0.532	< 0.0001	(***)	0.324	0.001	(**)	0.229	0.033	(*)
	8	0.477	< 0.0001	(***)	0.302	0.001	(**)	0.172	0.069	(-)
9	4	0.575	< 0.0001	(***)	0.437	< 0.0001	(***)	0.309	0.011	(*)
	5	0.599	< 0.0001	(***)	0.380	0.0002	(***)	0.212	0.041	(*)
	6	0.581	< 0.0001	(***)	0.350	0.001	(**)	0.191	0.054	(-)
	7	0.537	< 0.0001	(***)	0.327	0.001	(***)	0.176	0.0004	(***)
	8	0.486	< 0.0001	(***)	0.291	0.002	(**)	0.151	0.090	(-)
(c)										
6	4	0.330	< 0.0001	(***)	0.263	0.0002	(***)	0.416	0.032	(*)
	5	0.393	< 0.0001	(***)	0.213	0.001	(***)	0.322	0.069	(-)
	6	0.454	< 0.0001	(***)	0.214	0.001	(***)	0.337	0.061	(-)
	7	0.468	< 0.0001	(***)	0.256	0.000	(***)	0.426	0.030	(*)
	8	0.442	< 0.0001	(***)	0.278	< 0.0001	(***)	0.489	0.017	(*)
7	4	0.350	< 0.0001	(***)	0.260	0.0002	(***)	0.475	0.019	(*)
	5	0.426	< 0.0001	(***)	0.225	0.001	(**)	0.481	0.018	(*)
	6	0.484	< 0.0001	(***)	0.277	0.0001	(***)	0.472	0.020	(*)
	7	0.492	< 0.0001	(***)	0.317	< 0.0001	(***)	0.440	0.026	(*)
	8	0.485	< 0.0001	(***)	0.307	< 0.0001	(***)	0.448	0.024	(*)
8	4	0.373	< 0.0001	(***)	0.268	< 0.0001	(***)	0.577	0.007	(**)
	5	0.446	< 0.0001	(***)	0.263	0.0002	(***)	0.566	0.008	(**)
	6	0.492	< 0.0001	(***)	0.302	< 0.0001	(***)	0.473	0.019	(*)
	7	0.508	< 0.0001	(***)	0.303	< 0.0001	(***)	0.389	0.040	(*)
	8	0.504	< 0.0001	(***)	0.261	0.0002	(***)	0.273	0.099	(-)
9	4	0.394	< 0.0001	(***)	0.299	< 0.0001	(***)	0.631	0.004	(**)
	5	0.460	< 0.0001	(***)	0.282	< 0.0001	(***)	0.556	0.008	(**)
	6	0.510	< 0.0001	(***)	0.294	< 0.0001	(***)	0.417	0.032	(*)
	7	0.524	< 0.0001	(***)	0.266	0.0001	(***)	0.229	0.137	(-)
	8	0.519	< 0.0001	(***)	0.216	0.001	(***)	0.130	0.277	(-)

*p < 0.05, **p < 0.01, ***p < 0.001

**Table 2:** Spearman’s rank correlation between the effective reproduction numbers and the average mobility at transit stations considering the latencies. Mobility was averaged over 8 days with a latency of 6 days

		Spearman’s *ρ*	*p*-value	
Tokyo	W3	0.414	0.0012	(**)
	W4	0.557	< 0.0001	(***)
	W5	0.455	0.0025	(**)
Osaka	W3	0.7771	< 0.0001	(***)
	W4	0.688	< 0.0001	(***)
	W5	0.451	0.045	(*)
Aichi	W3	0.756	< 0.0001	(***)
	W4	0.493	0.0003	(***)
	W5	0.838	0.0013	(**)

^*^p < 0.05, ^**^p < 0.01, ^***^p < 0.001

Figure 2 shows the correlation between the ERN and average mobility at the transit stations during the upsurge and downsurge of DPC, wherein the duration and latency were set to 8 and 6 days, respectively. Note that the duration affected by the consecutive holidays in July (July 22–25) and the “Obon” holiday (August 7–15), which is a religious holiday in Japan, were excluded when deriving the regression line; the excluded durations were July 27 to August 6 and August 13 to August 28, according to the holidays in July and the “Obon” holiday, respectively. The period from January 3 to January 15 was also excluded, according to the New Year’s holiday. (In Fig. 2, only the primary date affected by the consecutive holidays were plotted.) A positive correlation was observed between the ERN and the time window averaged mobility during the upsurge. A negative correlation was observed during the down surge of positive cases.

Table 2 shows the Spearman rank correlation between the ERN and the mobility considering the latency. A positive correlation between the ERN and the time-averaged mobility at transit stations is significant. The correlation in Tokyo was less than that of Osaka and Aichi; however, a good correlation was observed as shown in Fig. 2. The larger the reduction rate of the mobility at the transit stations, the smaller the ERN.

**Fig. 3:**
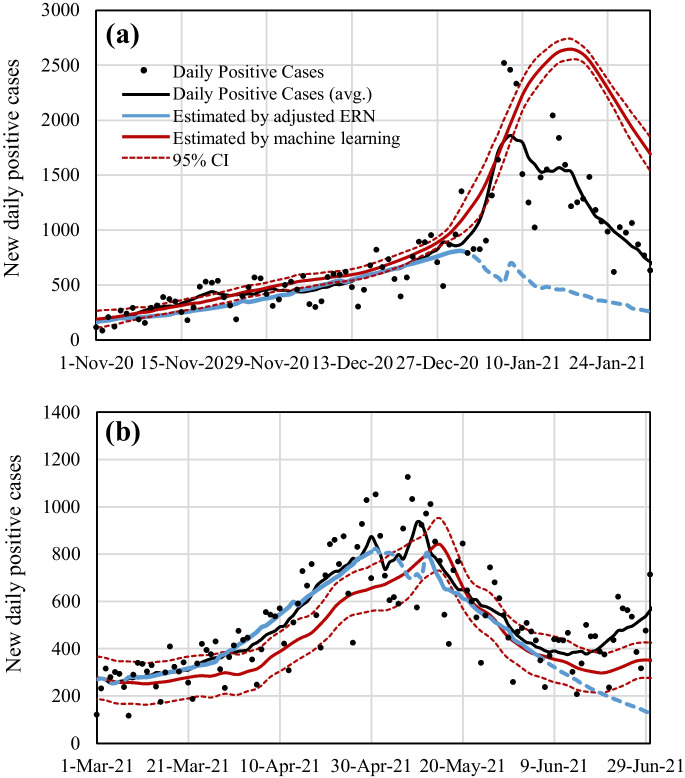
Daily positive cases (7-day average) estimated using the adjusted effective reproduction number (ERN) and LSTM of the **a** third (W3) and **b** fourth (W4) COVID-19 pandemic waves in Tokyo. The estimation was also conducted by machine learning [20]. The dashed part of the blue line corresponds to the estimation where the regression line approach is non-applicable (out of spread duration)

We then estimated the ERN by using the regression lines during the upsurge and downsurge respectively. Figure 3 demonstrates the estimation of DPC in terms of the ERN calculated by the regression lines in Fig. 2. For comparison, the estimation using the machine learning in [20] is also presented. DPCs after six days can be estimated using (1) with the ERN calculated by the regression lines using the mobility at the transit station. The estimation using the regression lines was in good agreement with the actual values during upsurge. The estimation including the consecutive holidays such as during the wave 3 (Fig. 3a) did not match the actual values due to different trends of time-averaged mobility at the transit station, as mentioned above.

## Discussion

Different factors were considered to associate with the ERN of COVID-19. In the multivariate analysis of epidemiological data processing, statistical associations with different factors are often discussed. Unlike such approaches, a surrogate was explored to directly correlate the ERN in three prefectures of Japan in a more feasible way based on our previous finding, which revealed the mobility at transit stations as the most dominant factor to determine DPC using machine learning forecasting [20]. The motivation for this investigation was that only a simple data processing was needed for understanding based on the newly reported DPC toward policy setting.

Considering the latency that is characterized by the incubation time and the delay until the patients reach a diagnosis, the time window was considered for the mobility averaged over, similarly to the ERN. From our data analysis, the duration and latency of the mobility were 8 (6–8) and 6 (6–7) days, respectively, to correlate with the ERN. The latency is in good agreement with the incubation time of 5.1 days (95% confidence interval (CI), 4.5–5.8 days) [30] and 5.8 days (95% CI, 5.0–6.7) [31] considering the latency to reporting in the healthcare facilities (0.5–2 days). The marginal difference may be attributable to the time required to move to the hospital or healthcare facility for diagnosis.

The time window chosen for mobility was comparable to that of the ERN. The uncertainty of this variation may be attributable to other cofactors, including the mobility at different places (e.g., parks), environmental conditions, and weekdays or weekends [20]. In [20], the effect of these other factors on new DPC was almost 6% on an average of over six prefectures in Japan. A similar conclusion was reached in a previous study [32]. The correlation for mobility averaged over different durations and latencies and the ERN in Tokyo was weaker than that in the remaining prefectures. From [20], the estimation accuracy of the new DPC in Tokyo was anticipated to be 62.7% and 83.0% from the mobility at the transit station and those including all places, respectively, even with machine learning. Conversely, these values were comparable to each other, i.e., 75% and 80% in Osaka and Aichi, respectively, even with the mobility at the transit station alone. Additionally, significant improvement was observed with other mobilities. This may be caused by the complexity of mobility in Tokyo, where multi-core cities or wards exist, unlike other prefectures (the population is approximately 12 million). In addition, several demographic and weather parameters were suggested to be related to the basic reproduction number, which would also serve as the variability of ERN [33]. They should also be an additional factor of uncertainty.

Using the surrogate identified from Table 2 (latency of 6 days and time window of 8 days), the relationship between the ERN and time-averaged mobility at transit stations was shown for the spread phase of Wuhan strain, Alpha, and Delta variants. As shown in Fig. 2 and Table 2, a good correlation was observed between these variants, especially for Osaka and Aichi. Even at the same mobility reduction rate, different ERNs were observed in Osaka and Aichi, suggesting that a target mobility reduction rate is different for policy settings to reduce the mobility to achieve an ERN lower than unity.

Moreover, from Fig. 2, the slope of regression lines is close to each other between the Wuhan strain, Alpha, and Delta variants. The ERN for the Alpha variants was 20% higher than that of the Wuhan strain in Osaka and Aichi when adjusted at the mobility. However, the straightforward comparison is not feasible for Alpha and Delta variants since the rate of fully vaccinated people increased during the spread of Delta variants. Figure 2 shows a few percent of the difference in the mobility-adjusted ERN between Alpha and Delta variants only in Osaka, whereas 5–20% in Aichi. Empirically assuming that the effective fully vaccinated population was 20–25% from July 20 to August 20, the mobility-adjusted ERN in Aichi and Osaka may be 20–50% higher than that in the Wuhan strain, without considering the break-through infection. The high ERN was observed during the consecutive holidays. A similar tendency was observed at the end of the spread duration of the third wave, which corresponds to the New Year holidays. Generally, the mobility (human behaviors) changes during such consecutive holidays resulted in the further spread of infection.

As shown in Figs. 4 and 5 of the Supplemental Document, this linear tendency was confirmed from London and Singapore for the selected spread duration. From these comparisons, in addition to the data in Fig. 2, the ERN highly depends on mobility; the mobility reduction is different to keep the ERN below 1. The ERN for the Alpha variants was 20–40% higher than that of the Wuhan strain in London. However, calculating the magnitude of the infectivity for the Delta variant was infeasible. According to previous studies in the UK [34], the infectivity of the Delta variant is higher than that of the Alpha variant (43–90%). This tendency is similar to that in Japan, but its magnitude was larger than that in Japan (20–50% from the Wuhan strain; 0–25% from the Alpha variant). The mobility adjustment, as we proposed in the present study, would be a possible reason for this difference. Spatial variability [35] and time variation [11] have been discussed in earlier studies. These may be attributable to mobility, as discussed in this study.

Using the regression line, the DPCs can be roughly estimated as shown in Fig. 3. In the period excluding the consecutive holidays such as during the fourth waves, the estimation by the regression line was in good agreement with the actual data. In contrast, our machine learninglstm models can estimate DPCs in 4 weeks after with 14.1% of accuracy. However, the estimation during the consecutive holidays, when people behavior are different than usual,  were difficult both by regression line and machine learning. The prediction by machine learning generally takes time to accumulate the data required for learning when the different situations from the past such as the appearance of a new variant [20]. The advantage of these two prediction ways can provide useful guidelines for early policy enforcement to reduce the mobility.

The limitation of this study includes the followings: (i) our approach is not intended to capture detailed phenomena, such as cluster infection reported at restaurants or nursing homes, but rather provide a rough guide for future mobility restrictions, and (ii) this linear tendency is observed in an urban area where primary mobility is characterized at train stations but not to areas where transportation is mainly via automobiles and other facilities (e.g., statistically insignificant for prefectures in Tohoku region). These points are not significant in metropolitan cities, where the pandemic is most crucial, and thus would be helpful for policymaking.

## Conclusion

In this study, the ERN is shown to close related to the mobility, when the time window is appropriately selected. The duration and latency were 8 and 6 days, respectively, from the analysis in three prefectures of Japan, which was consistent with London and Singapore. Linear correlation was observed between the time-averaged mobility and the ERN. Mobility adjustment is needed for proper comparison of viral infectivity in terms of ERN. This finding would be useful for other forecasting system, such as machine learning architecture designs. The mobility-adjusted ERN for the Alpha and Delta variants was 15–30% and 20–50% higher than that of the Wuhan strain for three prefectures in Japan, which was smaller than but consistent with the observed values in London. This simple metric can be a useful guideline for balanced policy enforcement on public movements toward viral infectivity reduction with minimum burden on daily-based activities and businesses.

## Supplementary Information

Below is the link to the electronic supplementary material.

Supplementary file1 (DOCX 127 KB)
