# Supplementary material for: The Effects of Time Window-Averaged Mobility on Effective Reproduction Number of COVID-19 Viral Variants in Urban Cities
Source: J Urban Health. 2022 Nov 29;100(1):29–39. doi: 10.1007/s11524-022-00697-5 (PMC9707419; doi:10.1007/s11524-022-00697-5)
Supplement: Supplementary file 1 — Supplementary file1 (DOCX 127 KB) [file 11524_2022_697_MOESM1_ESM.docx]

Appendix. Review of Related Studies

A.1. Effect of Mobility on Transmission

Straightforward discussion is infeasible because the sptial scale (city , state, or country levels) are different as well as the spread or the number of DPC is diffeernt. One notable and consistent findings are the importance of the mobility at workplace or transit stations, especially in urban cities. In [1], our estimation suggested that mobility at transit station is most correlated with new DPC ; LSTM architecture is used and thus considered as nonlinear regression. A city-level mobility index is shown to be correlated with COVID-19 transmission in Chinese cities with latency of 10 days (interquartile range 8–11 days) and correlation coefficients of 0.68±0.12 [2]. Workplace mobility is demonstrated to be statisitically significant to relate the spread in different countries [3]. At the early stage of the outbreak, the analysis model with the population mobility data can realize the short-term prediction of the pandemic spread [4].

A.2. Forecasting Period

Focusing on the forecasting period as well as accuracy, we briefly reviewed studies based on LSTM (long short term memory) network, one of the machine learning architecutre, because its accuracy is better than other appraochs (e.g., [5]). In [6], the estimation error of DPCs in India was < 20% after five days. Several neural networks were tested for estimating the number of DPCs in different cities of India [7]; an error was 3%–5% after one–three days. A rough estimation of the one-month prediction error is in the order of 20%–30% (from Fig. 6 in [8]). The difference between the predicted and actual error was <12.8% for a three-day prediction [9] using data fromRussia, Peru, and Iran obtained from January to July 2020. The accuracy of two-week estimation was 93.4%. In [10], data obtained from different European countries were compared using different models to forecast the two-week DPC. In our previous study [1], our proposed framework provided more accurate estimations than those provided by Google Cloud for four-week estimation. The two-week projected DPC can be estimated with an 81.6% accuracy for six prefectures in Japan. Overall, the forecasting period is rather variable in different studies, including input parameters, whereas no justification or rationale of the period is provided.

**References in Supplementary materials**

1. Rashed EA, Hirata A. One-Year Lesson: Machine Learning Prediction of COVID-19 Positive Cases with Meteorological Data and Mobility Estimate in Japan. Int J Environ Res Public Health. Multidisciplinary Digital Publishing Institute; 2021;18:5736.

2. Xi W, Pei T, Liu Q, Song C, Liu Y, Chen X, et al. Quantifying the time-lag effects of human mobility on the COVID-19 transmission: A multi-city study in China. IEEE Access. 2020;8:216752–61.

3. Hosseini MS, Masterangelo Gittler A. Factors influencing human mobility during the COVID-19 pandemic in selected countries of Europe and North America. 2020 IEEE Int Conf Big Data (Big Data). IEEE; 2020. p. 4866–72.

4. Wang R, Ji C, Jiang Z, Wu Y, Yin L, Li Y. A short-term prediction model at the early stage of the COVID-19 pandemic based on multisource urban data. IEEE Trans Comput Soc Syst. 2021;8:938–45.

5. Elsheikh AH, Saba AI, Panchal H, Shanmugan S, Alsaleh NA, Ahmadein M. Artificial Intelligence for Forecasting the Prevalence of COVID-19 Pandemic: An Overview. Healthcare. 2021;9:1614.

6. Tomar A, Gupta N. Prediction for the spread of COVID-19 in India and effectiveness of preventive measures. Sci Total Environ. Sci Total Environ; 2020;728.

7. Arora P, Kumar H, Panigrahi BK. Prediction and analysis of COVID-19 positive cases using deep learning models: A descriptive case study of India. Chaos, Solitons & Fractals. 2020;139:110017.

8. Kafieh R, Saeedizadeh N, Arian R, Amini Z, Serej ND, Vaezi A, et al. Isfahan and Covid-19: Deep spatiotemporal representation. Chaos, Solitons & Fractals. 2020;141:110339.

9. Wang P, Zheng X, Ai G, Liu D, Zhu B. Time series prediction for the epidemic trends of COVID-19 using the improved LSTM deep learning method: Case studies in Russia, Peru and Iran. Chaos, Solitons & Fractals. 2020;140:110214.

10. Kırbaş İ, Sözen A, Tuncer AD, Kazancıoğlu FŞ. Comparative analysis and forecasting of COVID-19 cases in various European countries with ARIMA, NARNN and LSTM approaches. Chaos, Solitons & Fractals. 2020;138:110015.

**Fig. 4** Mobility change (%) at transit stations and new daily confirmed cases in (a) Singapore and (b) London. Green, red, and blue colors correspond to the duration when the Wuhan strain, Alpha, and Delta variants were dominant, respectively.

**Fig. 5** Correlation between the mobility at transit stations and the effective reproduction number in (a) Singapore and (b) London. Mobility was averaged over 8 days with a latency of 6 days. Green, red, and blue colors correspond to the duration shown in Fig. 4, respectively.
